# Prognostic value of ^18^F-FDG-PET/CT in patients with nasopharyngeal carcinoma: a systematic review and meta-analysis

**DOI:** 10.18632/oncotarget.13934

**Published:** 2016-12-14

**Authors:** Jie Lin, Guozhu Xie, Guixiang Liao, Baiyao Wang, Miaohong Yan, Hui Li, Yawei Yuan

**Affiliations:** ^1^ Department of Radiation Oncology, Nanfang Hospital, Southern Medical University, Guangzhou, Guangdong, China; ^2^ Department of Radiation Oncology, Cancer Center of Guangzhou Medical University, Guangzhou, Guangdong, China; ^3^ Department of Radiation Oncology, Shenzhen people’s Hospital, Second Clinical Medicine College of Jinan University, Guangzhou, Guangdong, China

**Keywords:** ^18^F-FDG PET/CT, standardized uptake value, metabolic tumor volume, total lesion glycolysis, nasopharyngeal carcinoma

## Abstract

**Background:**

The prognostic role of ^18^F-fluorodeoxyglucose positron emission tomography CT (^18^F-FDG PET/CT) parameters is still controversial in nasopharyngeal carcinoma patients. We sought to perform a systematic review and meta-analysis to explore the prognostic value of maximal standardized uptake value (SUV_max_), metabolic tumor volume (MTV) and total lesion glycolysis (TLG) on event-free survival (EFS) and overall survival (OS) in nasopharyngeal carcinoma patients.

**Results:**

Fifteen studies comprising 1,938 patients were included in this study. The combined hazard ratios (HRs) for EFS were 2.63 (95%CI 1.71-4.05) for SUV_max_, 2.55 (95%CI 1.49-4.35) for MTV, and 3.32 (95%CI 1.23-8.95) for TLG. The pooled HRs for OS were 2.07 (95%CI 1.54-2.79) for SUV_max_, 3.86 (95%CI 1.85-8.06) for MTV, and 2.60 (95%CI 1.55-4.34) for TLG. The prognostic role of SUV_max_, MTV and TLG remained similar in the sub-group analyses.

**Methods:**

A systematic literature search was performed to identify studies which associated ^18^F-FDG PET/CT to clinical survival outcomes of nasopharyngeal carcinoma patients. The summarized HRs for EFS and OS were estimated by using fixed- or random-effect models according to heterogeneity between trials.

**Conclusions:**

The present meta-analysis confirms that high values of SUV_max_, MTV and TLG predicted a higher risk of adverse events or death in patients with nasopharyngeal carcinoma, despite clinically heterogeneous nasopharyngeal carcinoma patients and the various methods adopted between these studies.

## INTRODUCTION

Nasopharyngeal carcinoma (NPC) is a cancer deriving from the epithelial cells, which is covering the surface and lining the nasopharynx [1, 2]. Worldwidely speaking, 52.7% of new NPC cases were in World Health Organization (WHO) Western Pacific Region; the remainders are WHO South-East Asia, and Africa Region [3]. The age-standardized incidence in some ethnic groups is reported higher than others—eg, the Hmong in China, Bidayuh in Borneo, Inuits in the Artic, Nagas in northern India and Chamorro ethnic Polynesians [4]. The prognosis of NPC is related to the amount of conventional prognostic factors, such as TNM stage classification, history of smoking, clinical and molecular prognostic variables, and the raised plasma Epstein-Barr virus DNA is also one of the highlighted determinants of prognosis [2]. However, none of them can accurately assess the prognosis of patients in clinical practice.

In the early nineties, ^18^F-fluorodeoxyglucose positron emission tomography (^18^F-FDG PET) entered into clinical usage as a practical imaging technique in the regulation of neoplastic disorders, and it also applied in oncologic procedures such as TNM staging, restaging in progression and treatment efficacy assessment in different therapeutic process [5, 6]. In addition, various FDG parameters have been discussed during or after chemotherapy and radiotherapy as independent prognostic factors for outcome in numerous malignant tumor [6–8]. Standardized uptake value (SUV), a semi-quantitative parameter in ^18^F-FDG-PET/CT, is calculated as of the ratio of the FDG concentration to the weight-standardized injected dose in a region of interest (ROI) [9]. The most widely used parameter is SUV_max_, defined as the maximal SUV value in the ROI and is supposed to be a prognostic marker in some malignancies [6, 10–11]. Apart from SUV_max_, metabolic tumor volume (MTV) and total lesion glycolysis (TLG), as the tumor metabolic and volumetric parameter, are more widely applied in ^18^F-FDG-PET/CT recently [12]. MTV is the size of tumor tissues which is active ^18^F-FDG uptake, and TLG is the median SUV value in a region of interest multiplied by the MTV [13–15]. MTV and TLG might be utilized to represent the burthen of metabolically active lesion and tumor invasiveness in some malignancies [16].

However, a number of studies reported conflicting results of the prognostic values of SUV_max_, MTV and TLG in NPC patients [17–19]. Thus, this meta-analysis and systematic review was aimed at evaluating the prognostic values of ^18^F-FDG-PET/CT for survival outcomes in patients with NPC.

## RESULTS

### Search results

**Figure 1:**
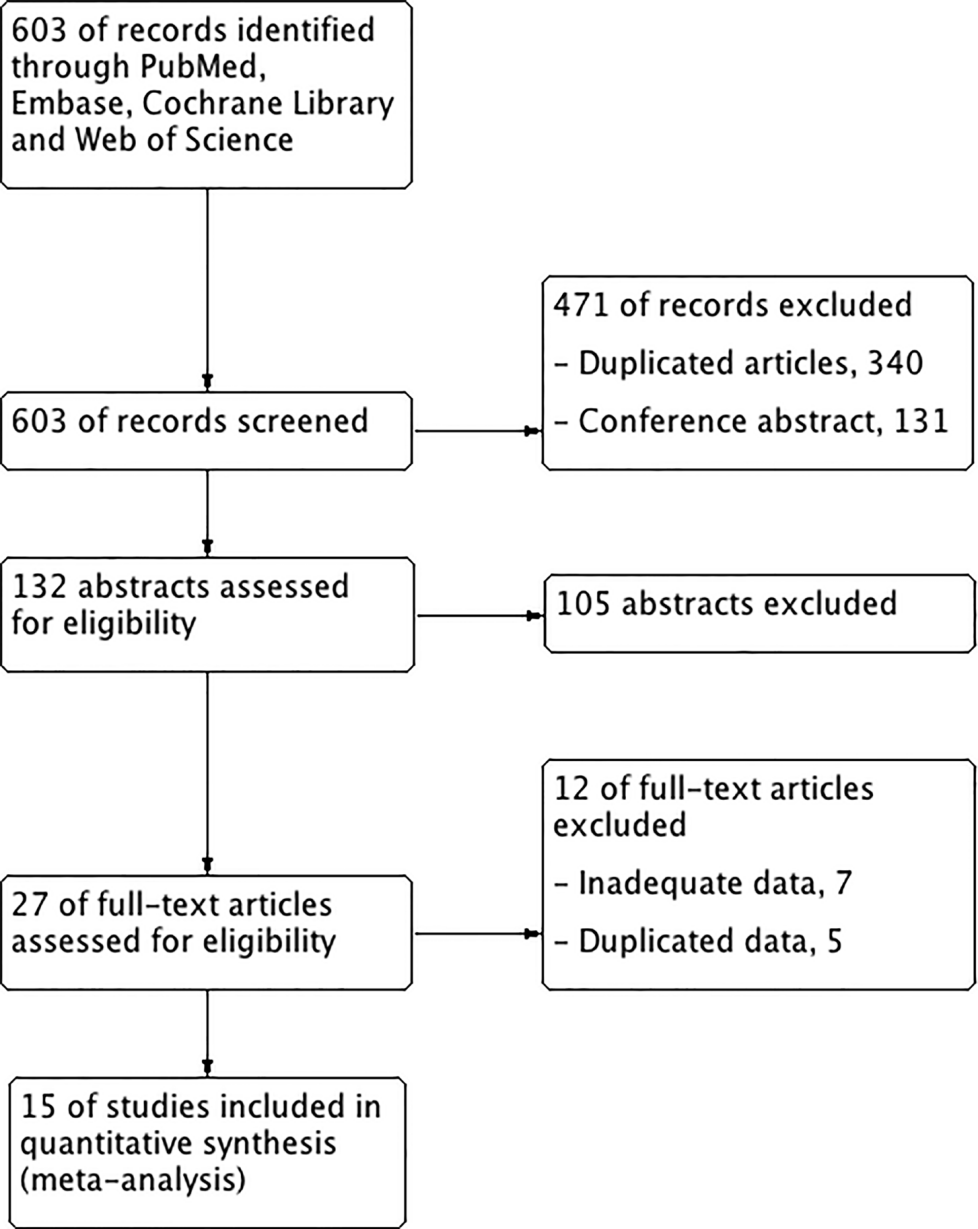
Flowchart of study selection

For primary retrieval, 603 articles were identified through 4 databases. The results were as follows: 336 articles from Embase, 169 articles from Web of Science, 98 articles in PubMed, and none from Cochrane Library. We firstly excluded the duplicates (*n* = 340) and conference abstracts (*n* = 131). Of the remaining, 105 articles were excluded according to the titles and abstracts, we included 27 potentially eligible articles from all databases and reviewed the full text. Of these articles, 7 were eliminated because the ln(hazard radio (HR)) and its variance of ^18^F-FDG-PET/CT parameters from NPC patients could not be extracted and calculated [20–25]; 4 were excluded because two author published 4 and 2 reports on the same population, respectively [26–28], [29]; and 1 article of overlapping patients was also excluded [18]. Finally, 1,938 patients of 15 studies published from 2008-2016 were eligible for this study (Figure 1) [17, 19, 30–42].

### Study characteristics and qualitative assessment

Table 1 shows the principal characteristics of the included studies. Nearly all of them were conducted in Asia, 6 studies in China, 4 studies in Taiwan, 3 studies in Korea, 1 in South Korea, and 1 in Egypt. 2 of them were of the prospective design and the remaining 13 studies were of the retrospective design. Of these studied 14 provided the sample size that ranged from 40 to 449 (median 70). The follow-up duration varied from 13.6 to 84.5 months (median 40.0 months).

**Table 1:** Characteristics of eligible studies included in the meta-analysis

Study	Year of publication	Patient source	Study period	Follow-up duration (range), months	Median age (range), years	Number of patients	TNM staging	End points provided	study design
Chan, S. C.[30]	2013	Taiwan	2006-2009	20.2(20-54)	NR	56	IV	ESF OS	Pro
Chan, W. K. S.[31]	2011	China	2007-2009	13.6±6.2(6.8-29.9)	4.8(16-78)	46	I-IV	EFS	Retro
Hsieh, T. C.[32]	2015	Taiwan	2004-2012	41.5	46(14-83)	174	II-IV	ESF OS	Retro
Hung, T. M.[33]	2013	Taiwan	2002-2008	64(3-108.2)	48.7(15-84)	371	I-IV	ESF OS	Retro
Lee, S. W.[34]	2008	Korea	2001-2003	40(8-58)	48(17-78)	41	I-IV	EFS	Retro
Liu, W. S. [35]	2012	Taiwan	1997-2003	56.4(31-81)	46.3(22-74)	75	I-IV	ESF OS	Retro
Moon, S. H.[19]	2015	Korea	2004-2009	40±17.6(9.0-71.6)	51.0±13.2(18-80)	44	I-IV	EFS	Retro
Shen, T.[36]	2015	China	2007-2013	18.09(0.62-55.88)	43.9(10-70)	194	I-IV	OS	Retro
Xiao, W. [17]	2015	China	2003-2008	84.5(6-118)	43(13-75)	179	I-IV	ESF OS	Pro
Xie, P.[37]	2010	China	2002-2004	61(9-69)	43(18-67)	62	III-IV	ESF OS	Retro
Yang, Z. [38]	2015	China	2006-2011	30.5(20-68)	52.5(28-70)	40	IV	ESF OS	Retro
Yoon, H. I. [39]	2016	Korea	2004-2013	47(8-127)	50(13-75)	97	III-IV	ESF OS	Retro
Yoon, Y. H. [40]	2014	South Korea	2006-2012	32.5(27.2-59.8)	48(21-69)	40	I-IV	OS	Retro
Zaghloul, H. A. [41]	2014	Egypt	2008-2012	39.7±10.9(14-58)	46(18-68)	70	II-IV	ESF OS	Retro
Zhang, Y. [42]	2016	China	2010-2012	49.5(3.37-67.9)	46(20-77)	449	I-IV	EFS	Retro

Table 2 shows the patterns of ^18^F-FDG PET scanning. Different scanners and various scanning protocols that patients received scans with were used in each study. The duration of fasting varied from 8 h to 4 h and not reported in 1 study. Serum blood glucose before injection ranged from 144-200 mg/dL and not reported in 6 studies. The injected dose varied from 296 to 555 MBq and the post-injection interval ranged from 45 to 70 min. Four threshold methods were used to calculate the cut-off values, including receiver-operating characteristics (ROCs) in 10 studies, minimum P value in 1 study, median value in 1 study, Contal and O’Quigley’s method in 1 study and not reported in 2 studies. Two threshold methods were applied to MTV and TLG for the segmentation of the primary NPC lesions. The fixed SUV of 2.5 was used in 4 articles [30, 38–40] and the isocontour method was used in 1 study [19]. The median cut-off point was 8.78 (5.0 to 15.6) for SUV_max_. The cut-off values of MTV varied from 28.9 to 110 cm^3^, and TLG values were between 249.1 and 764. The Newcastle-Ottawa Scale (NOS) scores are shown in Supplement Table 1 and all of the included studies have more than 6 scores.

**Table 2:** Methods of ^18^F-FDG PET imaging of the included studies

Study	PET scanners	Duration of fasting	Pre-injection blood glucose test	Post-injection interval	Dose of 18F-FDG	PET parameters	Determination of cut-off values	Tumor delineation	Cut-off values		
									SUV	MTV (cm^3^)	TLG
Chan, S. C.[30]	Discovery ST 16; GE Healthcare, Milwaukee, WI	6h	<150mg/dl	50-70min	370MBq	SUVmax, MTV, TLG	Minimum P value method	SUV2.5	12	110	560
Chan, W. K. S.[31]	Discovery VCT; 64MSCT, GE Healthcare Bio-Sciences Corp., Piscataway, NJ	6h	<144mg/dl	60min	4.8MBq/Kg	SUVmax	According to other study		7.5		
Hsieh, T. C.[32]	PET/CT-16 slice, Discovery STE; GE Medical Systems,Milwaukee, WI,	4h	NR	60min	370MBq	SUVmax	ROC curve		8.35		
Hung, T. M.[33]	CTI&Discovery ST; GE Healthcare	6h	NR	NR	370MBq	SUVmax	ROC curve		9.3		
Lee, S. W.[34]	Siemens/CTI, Knoxville,TN, USA	8h	NR	60min	15mCi	SUVmax	Median value		8		
Liu, W. S. [35]	ECAT ExactHR+, CTI, Knoxville, TN	6h	<150mg/dl	60min	370MBq	SUVmax	According to other study		5		
Moon, S. H.[19]	Discovery LS or Discovery STe, GE Healthcare, Milwaukee, WI, USA	6h	<200mg/dl	45-60min	5.55MBq/Kg	SUVmax, MTV, TLG	ROC curve	isocontour method	7.8	66	764
Shen, T.[36]	Discovery ST 16; GE,Healthcare, Little Chalfont, United Kingdom	NR	<200mg/dl	45-60min	5.55MBq/Kg	SUVmax	ROC curve		8.65		
Xiao, W. [17]	Discovery ST-16; General Electric Company	6h	NR	30-40min	4.4-7.4MBq/Kg	SUVmax	ROC curve		10.22		
Xie, P.[37]	Discovery LS PET/CT, GE	8h	NR	60min	5.55-7.4MBq/Kg	SUVmax	ROC curve		8		
Yang, Z. [38]	Knoxville, Tennessee,USA	4h	<10mmol/l	60min	7.4MBq/Kg	SUVmax, MTV, TLG	ROC curve	SUV2.5	15.6	28.9	249.1
Yoon, H. I. [39]	Discovery STE, GE Healthcare, or Biograph TruePoint 40, Siemens Healthcare, Malvern, PA	4h	NR	60min	370MBq	TLG	Contal and O’Quigley’s method	SUV2.5			322.7
Yoon, Y. H. [40]	Philips, Milpitas, CA	8h	<180mg/dl	45-60min	296-444MBq/Kg	SUVmax, MTV	ROC curve	SUV2.5	8.9	31.45	
Zaghloul, H. A. [41]	SOMATOMA,Project 10 CT Scanner	6h	<150mg/dl	60min	370MBq	SUVmax	ROC curve		10.3		
Zhang, Y. [42]	Discovery ST 16; GE Healthcare, Little Chalfont, UK	6h	<200mg/dl	45-60min	5.55MBq/Kg	SUVmax	ROC curve		10.45		

### Primary outcome: EFS

**Figure 2:**
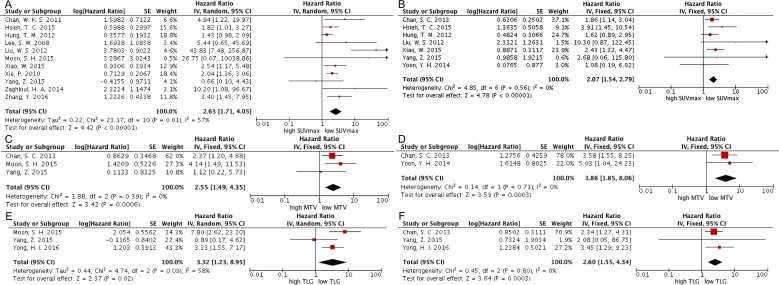
Forest plots of HR for EFS and OS with SUVmax (A, EFS; B, OS), MTV (C, EFS; D, OS) and TLG (E, EFS; F, OS) The Chi^2^ test is a measurement of heterogeneity. *P* < 0.05 indicates significant heterogeneity. Squares = individual study point estimates. Horizontal lines = 95%CIs. Rhombus = summarized estimate and its 95%CI. Fixed: fixed effect model. Random: random effect model.

**Figure 3:**
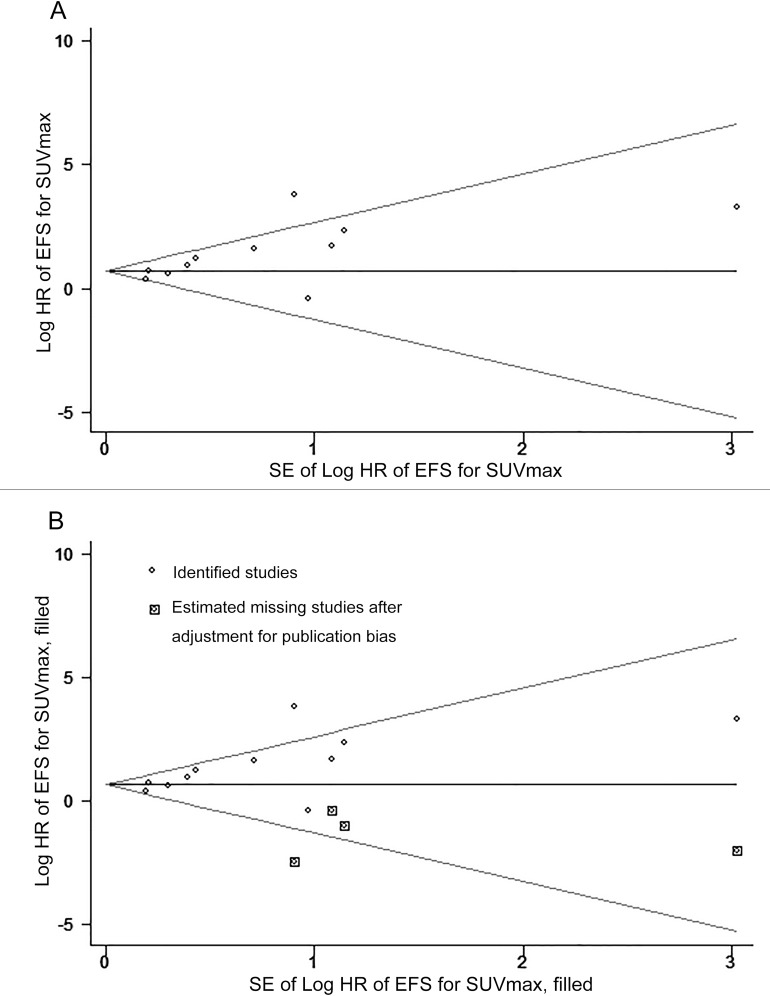
Funnel plots without (up column) and with (low column) trim and fill The pseudo 95% confidence interval (CI) is computed as part of the analysis that produced the funnel plot and corresponds to the expected 95%CI for a given standard error (SE). HR indicates hazard ratio.

11 studies were included to determine the association between SUV_max_ and event-free survival (EFS) and the combined data revealed that high SUV_max_ predict poor EFS (HR = 2.63; 95% confidence interval (CI) = 1.71-4.05, *P* < 0.00001; I^2^ = 57%) (Figure 2A). The potential publication bias was evaluated by two statistical test methods (Begg’s test and Egger’s test). The results (Begg’s test, z = 1.71, *P* = 0.087; Egger’s tests, *t* = 2.61, *P* = 0.028) indicated the possibility of publication bias owing to the statistically insignificant P value of Begg’s test. Therefore, herein we conducted a trim and fill analysis to ensure the reliability of the combined HR. The symmetrical funnel plot was demonstrated after the trim and fill analysis (Figure 3). When the hypothesized literatures were added, the results (pooled HR = 1.88; 95% CI = 1.52-2.33, *P* < 0.0001) of this sensitivity analysis still indicated that the correlation between SUV_max_ and EFS is significant. Also, we conducted sensitively analysis to further estimate the impact on the combined HRs. One study [35] were omitted, and an HR of 1.94 (1.56-2.43) was given a decreased I^2^ of 21% using a fixed-model.

On the one hand, 2 studies were included to analyze the prognostic value of MTV for EFS. Since no significant heterogeneity (χ^2^ = 1.88, *P* = 0.39; I^2^ = 0 %) was found among these studies, the HR was 2.55 (95%CI = 1.49 - 4.35, *P* = 0.0006) after using the fixed-effect model (Figure 2C). On the other hand, 3 studies were combined in the analysis of TLG for EFS. Significant heterogeneity (χ^2^ = 4.74, *P* = 0.09; I^2^ = 58 %) was found among these studies, so we used the random-effect model to calculate the HR (3.32, 95%CI = 1.23 - 8.95, *P* = 0.02) (Figure 2E). When the study of Yang, Z. et al. [38] was excluded, it reduced the heterogeneity from 58% to 36% (*P* = 0.21) and the pooled HR reached 4.41 (95%CI = 2.36-8.26).

According to the cut-off method, the threshold and the analysis method, we conducted the subgroup analyses. Among articles of SUV_max_, the HR of studies with cut-off values using ROC was 1.94 (95%CI: 1.47-2.58, *P* < 0.00001), and using other methods was 10.37 (95%CI: 2.52-42.69, *P* = 0.006). According to the median value of SUV_max_, the groups of cut-off values were divided into two subgroups—high (≥8.78) and low (< 8.78). Subgroup meta-analyses illustrated that the pooled HRs of SUV_max_ were 3.72 (95% CI: 1.01-13.67, *P* = 0.05) and 3.76 (95% CI = 1.76-8.04, *P* = 0.0006) for high and low cut-off value, respectively. For the analysis methods, the HR of studies using univariate analysis was 2.88 (95%CI = 1.44-5.79, *P* < 0.0001), and using multivariate analysis was 2.42 (95%CI = 1.62-3.62, *P* < 0.0001) (Table 3).

**Table 3:** Meta-analysis of the associations between ^18^F-FDG PET parameters and survival outcomes

Endpoint	Volumetric parameters	Factor	No. of studies	Heterogeneity test (I^2^, P)	Effect model	HR	95%CI of HR	Conclusion
EFS	SUV_max_	Cutoff method						
		ROC	8	20	fixed	1.94	1.47-2.58	significant
		Others	3	50	random	10.37	2.52-42.69	significant
		Threshold						
		≥8.78	5	90	random	3.72	1.01-13.67	significant
		<8.78	6	64	random	3.76	1.76-8.04	significant
		Analysis method						
		Univariate analysis	7	69	random	2.88	1.44-5.79	significant
		Multivariate analysis	4	3	fixed	2.42	1.62-3.62	significant
OS	SUV_max_	Cutoff method						
		ROC	5	0	fixed	2.13	1.45-3.12	significant
		Others	2	0	fixed	1.98	1.23-3.21	significant
		Threshold						
		≥8.78	5	0	fixed	1.89	1.38-2.60	significant
		<8.78	2	0	fixed	4.47	1.78-11.22	significant
		Analysis method						
		Univariate analysis	5	0	fixed	1.8	1.25-2.59	significant
		Multivariate analysis	2	0	fixed	2.77	1.65-4.66	significant

### Secondary outcome: OS

7 studies were included to assess the correlation between SUV_max_ and overall survival (OS). There was no significant heterogeneity (*P* = 0.56, χ^2^ = 4.85; I^2^ = 0 %) among these studies, so the fixed-effects model was applied to calculate the pooled HR (2.07, 95%CI = 1.54-2.79; *P* < 0.00001) (Figure 2B). At the same time, 2 studies were included to analyze the association between MTV and OS. High MTV significantly predicted the poor OS (HR = 3.86, 95% CI 1.85-8.06; *P* = 0.0003) without significant heterogeneity (χ^2^ = 0.14, *P* = 0.71; I^2^ = 0 %) (Figure 2D). 2 studies were included to evaluate TLG for OS and the result showed that high TLG significantly predicted the poor OS (HR = 2.60; 95% CI:1.55-4.34; I^2^ = 0%) without statistical heterogeneity (χ^2^ = 0.43, *P* = 0.51; I^2^ = 0%) among these studies (Figure 2F).

The results of the subgroup meta-analyses were demonstrated as following. Among the studies including SUV_max_, the HR of those with cutoff values using ROC method was 2.13 (95%CI: 1.45-3.12, *P* = 0.0001), and using other methods was 1.98 (95%CI: 1.23-3.21, *P* = 0.005); studies with high cut-off value had the HR of 1.89 (95%CI: 1.38-2.60, *P* < 0.0001), and the HR of those with low cut-off value was 4.47 (95%CI: 1.78-11.22, *P* = 0.001); studies using univariate analysis had the HR of 1.80 (95%CI: 1.25-2.59, P = 0.002), and the HR of those using multivariate analysis was 2.77 (95%CI: 1.65-4.66, *P* = 0.0001) (Table 3).

### Publication bias

Begg’s and Egger’s test were conducted to assess the publication bias. Funnel plots showing the correlations of SUV_max_ and OS (Supplement Figure 1), MTV and EFS (Supplement Figure 2), MTV and OS (Supplement Figure 3), TLG and EFS (Supplement Figure 4), TLG and OS (Supplement Figure 5), respectively. Visual observation of the Begg’s funnel plot and estimation of P values did not identify substantial asymmetry.

## DISCUSSION

Physicians sometimes face such an embarrassing situation that the standard therapies which are applied in a number of tumors, including NPC, are not effective, so how to reduce the toxicity of treatment failure and avoid unnecessary treatment becomes critical. [43]. From the literatures in recent years, not only the metabolic parameters of ^18^F-FDG PET/CT (SUV_max_, MTV and TLG) can be supposed to reflect the tumor biologic characteristics, but also can evaluate clinical prognosis [18, 38]. At present, SUV_max_ is considered to be the most frequently used value in diagnosis and therapeutic evaluation because of the high practicability, sensibility and efficiency [44–46]. Meanwhile, a poor prognostic value of SUV_max_ for head and neck cancer was reported in different staged and treated populations [47]. As is generally known, NPC is one of the most common types of head and neck cancer. There are some studies referring that SUV_max_ is one of the most important prognostic values of NPC patients [34]. However, SUV_max_ only demonstrates a simple tumor glucose metabolism within the lesion and cannot evaluate the heterogeneity of total tumor uptake. Recently, the prognostic value of MTV and TLG which are volumetric parameters is also pointed out in conference literatures [48–50]. Accordingly, we conducted a meta-analysis and revealed that higher values of SUV_max_, MTV and TLG, could predict a poor prognosis in NPC patients.

In this meta-analysis, the combined results demonstrated that SUV_max_ was a significant prognostic value for EFS and OS. But the association between SUV_max_ and survival outcomes may be affected by several confounding factors, so, the subgroup analysis of the statistical analysis method was conducted to validate the independent prognostic factor. Multivariate analysis is an effective method, which utilizes Cox proportional hazards model or logistic regression model to reduce bias from major confounders [51]. In our study, both univariate and multivariate subgroup of SUV_max_ were significant, so, it could be presumed that SUV_max_ might be one of the independent prognostic factors for survival outcomes. In addition, the methods to evaluate cut-off values are various in the included studies, such as ROS curve, minimal p-value approach and median value method, et al. Of all these methods, ROC was the most frequent and reasonable method to calculate the cut-off values in our meta-analysis. Although the use of other approaches including minimal p-value approach, might result in high false-positives, they were also reported widely applied in previous studies [52]. So subgroups stratified by the methods were conducted to evaluate the cut-off values.

It is still controversial that whether traditional imaging technique can predict NPC patients’ survival, because they only focus on tumor size. While MTV and TLG which were the volumetric parameters, could be utilized in metabolic analysis of radiotracer activity in tumor tissues and reflect the accurate tumor burden. Our study confirmed that high value of the volumetric parameters indicated poor EFS and OS, suggesting that ^18^F-FDG-PET/CT has vast prospect in predicting survival outcomes of NPC patients. To our knowledge, there were some articles studying on the parameters of PET of tumor or lymph nodes, but our study only focused on the parameters of tumor. Although 3 included articles [31, 33, 42] reported that SUV_max_ of lymph nodes was supposed to be an independent predictor of EFS or OS, there were no more statistics about MTV and TLG of lymph nodes for survival and we could not analyse them systematically. More studies are in need to further validate the findings.

We identified 22 previous meta-analyses assessing the clinical application of ^18^F-FDG-PET/CT in NPC and head and neck cancer by electronic search of PubMed (Table 4, Supplement Table 1). Only 4 of these literature were about NPC and they all analysed the accuracy of PET for residual and recurrent NPC or detected the lymph node and distant metastases [53–56]. As far as our information goes, our meta-analysis is the first to assess the prognostic values of ^18^F-FDG PET/CT parameters in NPC patients. Of the remaining studies on head and neck cancers, 14 studies analysed the diagnostic performance of PET for NPC [57–59], and distant metastasis [60–67], residual or recurrent disease [68–70] for head and neck cancers; 4 studies evaluated PET parameters for EFS, OS, disease-free survival (DFS) or loco-regional control using HRs, odds radios or risk radios [47, 71–73]. Pak. et al. suggested that the associations between high volumetric PET parameters (MTV and TLG) and the risk of adverse events, disease progression, or death were significant (i.e., an approximately 3-fold increase in the HR). In addition, they also demonstrated that high SUV_max_ was associated with worse EFS (HR = 1.83; 95% CI: 1.39-2.42) and worse OS (HR = 2.36; 95% CI, 1.48-3.77).

**Table 4:** Previous meta-analyses of ^18^F-FDG PET/CT in patients with nasopharyngeal carcinoma

Study	Year	Country	No. of studies	No. of patients	Classification	Effect size	Performance measure
Zhou, H.[53]	2016	China	23	1253	Diagnosis	Detecting residual or recurrent nasopharyngeal carcinoma	Sensitivity/specificity/likelihood ratios/odds ratios
Shen,G.[54]	2015	China	26	1203	Diagnosis	Detecting residual or recurrent nasopharyngeal carcinoma	Sensitivities/specificities/likelihood ratios
Shen,G.[55]	2014	China	20	2396	Staging	Detecting lymph node and distant metastases	Sensitivities/specificities/likelihood ratios
Chang, M. C.[56]	2013	Taiwan	8	1069	Staging	Detection of metastasis	Sensitivities/specificities/likelihood ratios

Heterogeneity was found in some analyses. On the one hand, some ^18^F-FDG-PET/CT imaging processes are significant contributors to heterogeneity —eg, fasting duration, pre-injected blood glucose level, post-injection interval and FDG doses. According to guidelines and protocols for ^18^F-FDG PET imaging [74–76], it recommend that duration of fasting should be at least 4h, pre-injection blood glucose can be level less than 200 mg/dL and a post-injection interval must be less than 75 min. The heterogeneity of the results was acceptable since the values were within normal range. On the other hand, the PET imaging thresholds found obviously between the studies can also induce the heterogeneity, which could be interpreted by various influence factors, such as the PET machine types, treatment protocol variations, different scanning executions, diversity of patient cohorts and variations of institutional technical [77–79]. A subgroup analysis of SUV_max_ was performed based on median values, however, the cut-off values and ^18^F-FDG PET scanning techniques being used in these studies were different and the number of studies was too small to apply as groups.

Moreover, this study indeed has a few limitations. Firstly, the quality of the included studies can also be taken into account as a limitation of our study. Although all of the included studies were evaluated by NOS scores and considered as high quality, we included only 2 prospective studies, some studies still lacked partial details of patients and data of ^18^F-FDG PET scan. Further prospective studies combining survival rate of NPC and PET parameters are needed. Secondly, we only included the English articles so that the potential effect of language bias should not be ignored. Thirdly, only published studies had been included when we searched the electronic databases, so the publication bias could not be excluded, even though the Begg’s test was conducted and did not suggest clear evidence of it. Moreover, the final result of our trim and fill sensitivity analysis was not affected after incorporating the hypothetical missing literatures, which demonstrates that our analysis was reliable. In addition, the included studies of this meta-analysis are almost in Asia, only one [41] in Africa, none in Europe and other continents. Because the incident of NPC is high in these regions and countries and it may cause the bias of the race of humans. Finally, it may lead to imprecision that Engauge Digitizer was used to extract the data of HRs from survival curves indirectly. Nonetheless, some recent clinical studies [79, 80] supported the validity of the main results in our study.

## MATERIALS AND METHODS

### Search strategies

We systematically searched PubMed, Embase, Cochrane Libraryand Web of Science with no restriction on language and date of publication. The last search was conducted on July 4, 2016, using the following terms: (“nasopharynx cancer” or “nasopharyngeal carcinoma” or “nasopharyngeal cancer” or “nasopharynx carcinoma”) and (“positron emission tomography” or “positron emission tomography-computed tomography” or “positron emission tomography computed tomography” or “PET” or “PET-CT” or “PET CT” or “PET/CT” or “fluorodeoxyglucose” or “FDG”) and (“prognostic” or “prognosis” or “predictive” or “survival” or “outcome”).

### Inclusion and exclusion criteria

All studies in the meta-analysis should meet the following criteria: (1) patients diagnosed with nasopharyngeal carcinoma pathologically; (2) case control study or cohort; (3) at least once ^18^F-FDG PET scan before or/and in treatment (4) referring to PET-CT prognostic value, such as OS, DFS, EFS, progress-free survival (PFS) and disease metastasis-free survival (DMFS) and event-free survival (EFS); (5) providing the HRs and 95%CIs and other useful information; (6) were in language of English. Articles were excluded by following criteria: (1) based on the study of animals or cells; (2) comment letters, case report, conference abstracts; (3) had not enough data to calculate the HRs and 95%CIs; (4) the research is limited in PET-CT of diagnosis and tumor staging, not provide prognostic parameters. (5) less than 10 patients. When articles recruiting overlapping patients were detected, only the most complete or recent studies include. Two authors (J Lin and MH Yan) independently evaluated the literature review for eligibility. Disagreements were under discussion and adjudicated by the corresponding author (GZ Xie).

### Data extraction

Two authors (J Lin and H Li) performed the data extraction independently from the publications. A Microsoft Excel sheet was designed to collect the following items: (1) Basic information of study including author names, year of publication, study period, follow-up duration, study design; (2) Details of patient and tumor including patient source, number, median age, TNM staging and end points provided; (3) Data of ^18^F-FDG-PET scan and parameters including PET scanners, duration of fasting before FDG injection, pre-injection blood glucose test, radiation doses of FDG, post-injection interval, the method of determination of cut-off values, PET parameters, tumor delineation and cut-off values of SUV_max_, MTV, TLG.

### Quality assessment

According to the Newcastle-Ottawa Scale criteria (http://www.ohri.ca/programs/clinical_epidemiology/oxford.asp), two investigators (J Lin and GX Liao) independently assessed the quality of the potentially included studies. The NOS criteria are scored based on three items: subject selection, comparability of subject and outcome (cohort studies) or exposure (case control). For quality assessment, each item had three scores and a total of scores varied from 0 (lowest) to 9 (highest). During this process, we suggested that studies with scores ≥6 were rated as high quality studies and scores less than 6 were excluded in this meta-analysis and discrepancies were resolved by consensus (Supplement Table 1).

### Statistical analysis

In this meta-analysis, disease-free survival, progression-free survival, disease metastasis-free survival in the included studies were merged and redefined as EFS. The primary endpoint was EFS, defined as the time from initiation of therapy until recurrence or metastasis [43]. The secondary outcome was OS, which was measured from the date of initiation of therapy to the date of death from any cause. The impact of ^18^F-FDG PET parameters on survival outcomes was measured by the effective size of the HR. HR values of included study were extracted using the following methodology suggested by Parmar et al. [81] and Tierney et al. [82] HR values and its 95% CIs from included studies could be directly extracted if the original data was supplied by the authors. Otherwise, P values of the log-rank test, number of events, and total number of patients in each group were extracted to estimate the HR indirectly; or, we extracted the HRs from survival curves. We presumed that patients were censored at a constant rate during the follow-up, and the Kaplan-Meier curves were read by Engauge Digitizer (version 8.2 for Mac; http://digitizer.sourceforge.net) to reconstruct the HR estimate and its variance. An observed HR>1 indicated a worse prognosis in patients with high parameter value and HR < 1 suggested a better prognosis. Heterogeneity between studies was evaluated by Chi-square test and I^2^ statistics, following recommendation of Cochrane Handbook (http://handbook.cochrane.org/). If P-value was >0.1 or/and I^2^ < 50%, indicating there was no or moderate heterogeneity, a fixed-effects model was used; otherwise, the random-effects model was used. The analyses described above were conducted by Review Manager (RevMan, version 5.3; The Nordic Cochrane Centre, The Cochrane Collaboration). Begg’s funnel test and Egger’s test were made for testing publication bias by STATA version 12.0 (STATA Corp., College Station, TX). It is considered statistically significant when a P-value is less than 0.05.

## CONCLUSION

This meta-analysis demonstrated that NPC patients with a high SUV_max_, MTV or TLG of ^18^F-FDG-PET/CT are at higher risk for adverse events or death, despite clinically heterogeneous NPC patients and the various methods adopted between studies. ^18^F-FDG-PET/CT can be used for risk stratification in disease control and survival. Future multi-center studies are needed to validate our findings and further explore the significant prognosis value of other ^18^F-FDG PET/CT parameters in prolonging survival of NPC patients.

## SUPPLEMENTARY MATERIALS TABLES





## References

[1] Brennan B (2006). Nasopharyngeal carcinoma. Orphanet J Rare Dis.

[2] Chua ML, Wee JT, Hui EP, Chan AT (2016). Nasopharyngeal carcinoma. Lancet.

[3] Ferlay J, Soerjomataram I, Ervik M, Dikshit R, Eser S, Mathers C, Rebelo M, Parkin DM, Forman D, Bray F http://globocan.iarc.fr/today.

[4] Wee JT, Ha TC, Loong SL, Qian CN (2010). Is nasopharyngeal cancer really a “Cantonese cancer”?. Chinese journal of cancer.

[5] Czernin J, Phelps ME (2002). Positron emission tomography scanning: current and future applications. Annu Rev Med.

[6] Gallamini A, Zwarthoed C, Borra A (2014). Positron Emission Tomography (PET) in Oncology. Cancers (Basel).

[7] Chung MK, Jeong HS, Park SG, Jang JY, Son YI, Choi JY, Hyun SH, Park K, Ahn MJ, Ahn YC, Kim HJ, Ko YH, Baek CH (2009). Metabolic tumor volume of [18F]-fluorodeoxyglucose positron emission tomography/computed tomography predicts short-term outcome to radiotherapy with or without chemotherapy in pharyngeal cancer. Clin Cancer Res.

[8] Paidpally V, Chirindel A, Chung CH, Richmon J, Koch W, Quon H, Subramaniam RM (2014). FDG volumetric parameters and survival outcomes after definitive chemoradiotherapy in patients with recurrent head and neck squamous cell carcinoma. AJR Am J Roentgenol.

[9] Strauss LG, Conti PS (1991). The applications of PET in clinical oncology. Journal of nuclear medicine.

[10] Doi H, Kitajima K, Fukushima K, Kawanaka Y, Mouri M, Yamamoto S, Ishikura R, Terada T, Noguchi K, Hirota S (2016). SUVmax on FDG-PET is a predictor of prognosis in patients with maxillary sinus cancer. Jpn J Radiol.

[11] Rahman T, Tsujikawa T, Yamamoto M, Chino Y, Shinagawa A, Kurokawa T, Tsuchida T, Kimura H, Yoshida Y, Okazawa H (2016). Different Prognostic Implications of 18F-FDG PET Between Histological Subtypes in Patients With Cervical Cancer. Medicine (Baltimore).

[12] Tylski P, Stute S, Grotus N, Doyeux K, Hapdey S, Gardin I, Vanderlinden B, Buvat I (2010). Comparative assessment of methods for estimating tumor volume and standardized uptake value in (18)F-FDG PET. Journal of nuclear medicine.

[13] Boellaard R, Delgado-Bolton R, Oyen WJG, Giammarile F, Tatsch K, Eschner W, Verzijlbergen FJ, Barrington SF, Pike LC, Weber WA, Stroobants S, Delbeke D, Donohoe KJ (2015). FDG PET/CT: EANM procedure guidelines for tumour imaging: version 2.0. Eur J Nucl Med Mol I.

[14] Lee JW, Kang CM, Choi HJ, Lee WJ, Song SY, Lee JH, Lee JD (2014). Prognostic Value of Metabolic Tumor Volume and Total Lesion Glycolysis on Preoperative (1)(8)F-FDG PET/CT in Patients with Pancreatic Cancer. Journal of nuclear medicine.

[15] Husby JA, Reitan BC, Biermann M, Trovik J, Bjorge L, Magnussen IJ, Salvesen OO, Salvesen HB, Haldorsen IS (2015). Metabolic Tumor Volume on 18F-FDG PET/CT Improves Preoperative Identification of High-Risk Endometrial Carcinoma Patients. Journal of nuclear medicine.

[16] Davison J, Mercier G, Russo G, Subramaniam RM (2013). PET-based primary tumor volumetric parameters and survival of patients with non-small cell lung carcinoma. AJR American journal of roentgenology.

[17] Xiao W, Xu A, Han F, Lin X, Lu L, Shen G, Huang S, Fan W, Deng X, Zhao C (2015). Positron emission tomography-computed tomography before treatment is highly prognostic of distant metastasis in nasopharyngeal carcinoma patients after intensity-modulated radiotherapy treatment: a prospective study with long-term follow-up. Oral oncology.

[18] Chang KP, Tsang NM, Liao CT, Hsu CL, Chung MJ, Lo CW, Chan SC, Ng SH, Wang HM, Yen TC (2012). Prognostic significance of 18F-FDG PET parameters and plasma Epstein-Barr virus DNA load in patients with nasopharyngeal carcinoma. J Nucl Med.

[19] Moon SH, Choi JY, Lee HJ, Son YI, Baek CH, Ahn YC, Ahn MJ, Park K, Kim BT (2015). Prognostic value of volume-based positron emission tomography/computed tomography in patients with nasopharyngeal carcinoma treated with concurrent chemoradiotherapy. Clin Exp Otorhinolaryngol.

[20] Chen WH, Tang LQ, Zhang L, Chen QY, Guo SS, Liu LT, Fan W, Zhang X, Guo L, Zhao C, Cao KJ, Qian CN, Guo X (2015). Combining plasma Epstein-Barr virus DNA and nodal maximal standard uptake values of 18F-fluoro-2-deoxy-D-glucose positron emission tomography improved prognostic stratification to predict distant metastasis for locoregionally advanced nasopharyngeal carcinoma. Oncotarget.

[21] Su M, Zhao L, Wei H, Lin R, Zhang X, Zou C (2015). 18F-fluorodeoxyglucose positron emission tomography for predicting tumor response to radiochemotherapy in nasopharyngeal carcinoma. Strahlenther Onkol.

[22] Lin P, Min M, Lee M, Holloway L, Forstner D, Bray V, Xuan W, Chicco A, Fowler A (2016). Prognostic utility of 18F-FDG PET-CT performed prior to and during primary radiotherapy for nasopharyngeal carcinoma: Index node is a useful prognostic imaging biomarker site. Radiother Oncol.

[23] Murphy JD, La TH, Chu K, Quon A, Fischbein NJ, Maxim PG, Graves EE, Loo BW, Le QT (2011). Postradiation metabolic tumor volume predicts outcome in head-and-neck cancer. Int J Radiat Oncol Biol Phys.

[24] Xie P, Yue JB, Zhao HX, Sun XD, Kong L, Fu Z, Yu JM (2010). Prognostic value of 18F-FDG PET-CT metabolic index for nasopharyngeal carcinoma. J Cancer Res Clin Oncol.

[25] Yen TC, Lin CY, Wang HM, Huang SF, Liao CT, Kang CJ, Ng SH, Chan SC, Fan KH, Chen IH, Lin WJ, Cheng AJ, Chang JT (2006). 18F-FDG-PET for evaluation of the response to concurrent chemoradiation therapy with intensity-modulated radiation technique for Stage T4 nasopharyngeal carcinoma. Int J Radiat Oncol Biol Phys.

[26] Chan SC, Kuo WH, Wang HM, Chang JT, Lin CY, Ng SH, Hsu CL, Chang KP, Liao CT, Lin YJ, Yen TC (2013). Prognostic implications of post-therapy (18)F-FDG PET in patients with locoregionally advanced nasopharyngeal carcinoma treated with chemoradiotherapy. Ann Nucl Med.

[27] Chan SC, Chang JT, Lin CY, Ng SH, Wang HM, Liao CT, Chang CJ, Lin SY, Yen TC (2011). Clinical utility of 18F-FDG PET parameters in patients with advanced nasopharyngeal carcinoma: predictive role for different survival endpoints and impact on prognostic stratification. Nucl Med Commun.

[28] Chan SC, Chang JT, Wang HM, Lin CY, Ng SH, Fan KH, Chin SC, Liao CT, Yen TC (2009). Prediction for distant failure in patients with stage M0 nasopharyngeal carcinoma: the role of standardized uptake value. Oral oncology.

[29] Shi Q, Yang Z, Zhang Y, Hu C (2014). Adding maximum standard uptake value of primary lesion and lymph nodes in 18F-fluorodeoxyglucose PET helps predict distant metastasis in patients with nasopharyngeal carcinoma. PloS one.

[30] Chan SC, Hsu CL, Yen TC, Ng SH, Liao CT, Wang HM (2013). The role of 18F-FDG PET/CT metabolic tumour volume in predicting survival in patients with metastatic nasopharyngeal carcinoma. Oral oncology.

[31] Chan WKS, Kwong DLW, Yeung DWC, Huang B, Khong PL (2011). Prognostic impact of standardized uptake value of F-18 FDG PET/CT in nasopharyngeal carcinoma. Clin Nucl Med.

[32] Hsieh TC, Hsieh CY, Yang TY, Chen TT, Lin CY, Lin CC, Hua CH, Chiu CF, Yeh SP, Sher YP (2015). [18F]-Fluorodeoxyglucose Positron Emission Tomography Standardized Uptake Value as a Predictor of Adjuvant Chemotherapy Benefits in Patients With Nasopharyngeal Carcinoma. Oncologist.

[33] Hung TM, Wang HM, Kang CJ, Huang SF, Liao CT, Chan SC, Ng SH, Chen IH, Lin CY, Fan KH, Chang JT (2013). Pretreatment (18)F-FDG PET standardized uptake value of primary tumor and neck lymph nodes as a predictor of distant metastasis for patients with nasopharyngeal carcinoma. Oral oncology.

[34] Lee SW, Nam SY, Im KC, Kim JS, Choi EK, Ahn SD, Park SH, Kim SY, Lee BJ, Kim JH (2008). Prediction of prognosis using standardized uptake value of 2- [(18)F] fluoro-2-deoxy-d-glucose positron emission tomography for nasopharyngeal carcinomas. Radiother Oncol.

[35] Liu WS, Wu MF, Tseng HC, Liu JT, Weng JH, Li YC, Lee JK (2012). The role of pretreatment FDG-PET in nasopharyngeal carcinoma treated with intensity-modulated radiotherapy. Int J Radiat Oncol Biol Phys.

[36] Shen T, Tang LQ, Luo DH, Chen QY, Li PJ, Mai DM, Guo SS, Liu LT, Qian CN, Guo X, Zeng MS, Mo HY, Mai HQ (2015). Different prognostic values of plasma Epstein-Barr virus DNA and maximal standardized uptake value of 18F-FDG PET/CT for nasopharyngeal carcinoma patients with recurrence. PloS one.

[37] Xie P, Yue JB, Fu Z, Feng R, Yu JM (2010). Prognostic value of 18F-FDG PET/CT before and after radiotherapy for locally advanced nasopharyngeal carcinoma. Ann Oncol.

[38] Yang Z, Shi Q, Zhang Y, Pan H, Yao Z, Hu S, Shi W, Zhu B, Zhang Y, Hu C (2015). Pretreatment (18)F-FDG uptake heterogeneity can predict survival in patients with locally advanced nasopharyngeal carcinoma—a retrospective study. Radiat Oncol.

[39] Yoon HI, Kim KH, Lee J, Roh YH, Yun M, Cho BC, Lee CG, Keum KC (2016). The Clinical Usefulness of (18)F-Fluorodeoxyglucose Positron Emission Tomography (PET) to Predict Oncologic Outcomes and PET-Based Radiotherapeutic Considerations in Locally Advanced Nasopharyngeal Carcinoma. Cancer Res Treat.

[40] Yoon YH, Lee SH, Hong SL, Kim SJ, Roh HJ, Cho KS (2014). Prognostic value of metabolic tumor volume as measured by fluorine-18-fluorodeoxyglucose positron emission tomography/computed tomography in nasopharyngeal carcinoma. Int Forum Allergy Rhinol.

[41] Zaghloul HA, Khedr GA, Rostom Y, Refaat T (2014). The Predictive Value of Pretreatment 18-F-FDG-PET-CT in Locally Advanced Nasopharyngeal Cancer Patients Treated Definitively with Induction Chemotherapy Followed by Concurrent Chemo-Radiotherapy. J Nucl Med Radiat Ther.

[42] Zhang Y, Li WF, Mao YP, Zhou GQ, Peng H, Sun Y, Liu Q, Chen L, Ma J (2016). Establishment of an integrated model incorporating standardised uptake value and N-classification for predicting metastasis in nasopharyngeal carcinoma. Oncotarget.

[43] Zhao Q, Feng Y, Mao X, Qie M (2013). Prognostic value of fluorine-18-fluorodeoxyglucose positron emission tomography or PET-computed tomography in cervical cancer: a meta-analysis. International journal of gynecological cancer.

[44] Kinahan PE, Fletcher JW (2010). Positron emission tomography-computed tomography standardized uptake values in clinical practice and assessing response to therapy. Semin Ultrasound CT MR.

[45] Chen L, Zhang N, Wang Y, Xian W, Hu W, Wei G (2015). Value of FDG PET-CT associated with pathology in diagnosing residual tumor in patients with nasopharyngeal carcinoma after radiotherapy. [Article in Chinese]. Zhonghua Zhong Liu Za Zhi.

[46] Lin CC, Hsieh TC, Chen TT, Hsieh CY, Lin CY, Huang HH, Bai LY, Lin PH, Chiu CF, Yeh SP, Liao YM, Lo WC (2013). Predictive values of (18)f-FDG PET standardized uptake value for adjuvant chemotherapy in patients with nasopharyngeal carcinoma. J Clin Oncol.

[47] Zhang B, Li X, Lu X (2010). Standardized uptake value is of prognostic value for outcome in head and neck squamous cell carcinoma. Acta Otolaryngol.

[48] Pilar A, Laskar SG, Budrukkar A, Gupta T, Murthy V, Agarwal JP (2016). Can PET-CT based parameters replace traditional TNM based prognostication in carcinoma nasopharynx treated with definitive chemo radiotherapy?. Eur J Cancer.

[49] Ghosh Laskar S, Pilar A, Purandare N, Rangarajan V, Budrukkar A, Gupta T, Murthy V (2015). Clinical impact of metabolic and anatomic imaging in nasopharyngeal carcinoma treated with chemoradiotherapy. Radiother Oncol.

[50] Lan X, Tian Y, Zhang Y (2014). Prognostic predictive value of total lesion glycolysis from 18F-FDG PET/CT in nasopharyngeal carcinoma patients after comprehensive therapy. Eur J Nucl Med Mol I.

[51] Jupiter DC (2015). Causal diagrams and multivariate analysis III: confound it!. J Foot Ankle Surg.

[52] Altman DG, Lausen B, Sauerbrei W, Schumacher M (1994). Dangers of using “optimal” cutpoints in the evaluation of prognostic factors. J Natl Cancer I.

[53] Zhou H, Shen G, Zhang W, Cai H, Zhou Y, Li L (2016). 18F-FDG PET/CT for the Diagnosis of Residual or Recurrent Nasopharyngeal Carcinoma After Radiotherapy: A Metaanalysis. J Nucl Med.

[54] Shen G, Zhou L, Jia Z, Zhang W, Wang Q, Deng H (2015). Meta-analysis of PET/CT for diagnosis of residual/recurrent nasopharyngeal carcinoma. [Article in Chinese]. Journal of clinical otorhinolaryngology, head, and neck surgery.

[55] Shen G, Zhang W, Jia Z, Li J, Wang Q, Deng H (2014). Meta-analysis of diagnostic value of 18F-FDG PET or PET/CT for detecting lymph node and distant metastases in patients with nasopharyngeal carcinoma. The British journal of radiology.

[56] Chang MC, Chen JH, Liang JA, Yang KT, Cheng KY, Kao CH (2013). Accuracy of whole-body FDG-PET and FDG-PET/CT in M staging of nasopharyngeal carcinoma: a systematic review and meta-analysis. Eur J Radiol.

[57] Xiao Y, Chen Y, Shi Y, Wu Z (2015). The value of fluorine-18 fluorodeoxyglucose PET/MRI in the diagnosis of head and neck carcinoma: a meta-analysis. Nucl Med Commun.

[58] Rohde M, Dyrvig AK, Johansen J, Sorensen JA, Gerke O, Nielsen AL, Hoilund-Carlsen PF, Godballe C (2014). 18F-fluoro-deoxy-glucose-positron emission tomography/computed tomography in diagnosis of head and neck squamous cell carcinoma: a systematic review and meta-analysis. Eur J Cancer.

[59] Pasamontes Pingarron JA, Cabrera Martin MN, Delgado Bolton RC, Fernandez Perez C, Carreras Delgado JL, Scola Yurrita B (2008). [Systematic review and meta-analysis of diagnostic accuracy of 18F-FDG PET in suspected recurrent head and neck cancer]. Acta otorrinolaringologica espanola.

[60] Sun R, Tang X, Yang Y, Zhang C (2015). (18)FDG-PET/CT for the detection of regional nodal metastasis in patients with head and neck cancer: a meta-analysis. Oral oncology.

[61] Yongkui L, Jian L, Wanghan Jingui L (2013). 18FDG-PET/CT for the detection of regional nodal metastasis in patients with primary head and neck cancer before treatment: a meta-analysis. Surg Oncol.

[62] Yi X, Fan M, Liu Y, Zhang H, Liu S (2013). 18 FDG PET and PET-CT for the detection of bone metastases in patients with head and neck cancer. A meta-analysis. J Med Imaging Radiat Oncol.

[63] Xu G, Li J, Zuo X, Li C (2012). Comparison of whole body positron emission tomography (PET)/PET-computed tomography and conventional anatomic imaging for detecting distant malignancies in patients with head and neck cancer: a meta-analysis. Laryngoscope.

[64] Xu GZ, Zhu XD, Li MY (2011). Accuracy of whole-body PET and PET-CT in initial M staging of head and neck cancer: a meta-analysis. Head & neck.

[65] Xu GZ, Guan DJ, He ZY (2011). (18)FDG-PET/CT for detecting distant metastases and second primary cancers in patients with head and neck cancer. A meta-analysis. Oral oncology.

[66] Gupta T, Master Z, Kannan S, Agarwal JP, Ghsoh-Laskar S, Rangarajan V, Murthy V, Budrukkar A (2011). Diagnostic performance of post-treatment FDG PET or FDG PET/CT imaging in head and neck cancer: a systematic review and meta-analysis. Eur J Nucl Med Mol Imaging.

[67] Kyzas PA, Evangelou E, Denaxa-Kyza D, Ioannidis JP (2008). 18F-fluorodeoxyglucose positron emission tomography to evaluate cervical node metastases in patients with head and neck squamous cell carcinoma: a meta-analysis. J Natl Cancer Inst.

[68] Cheung PK, Chin RY, Eslick GD (2016). Detecting Residual/Recurrent Head Neck Squamous Cell Carcinomas Using PET or PET/CT: Systematic Review and Meta-analysis. Otolaryngology—head and neck surgery.

[69] Gao S, Li S, Yang X, Tang Q (2014). 18FDG PET-CT for distant metastases in patients with recurrent head and neck cancer after definitive treatment. A meta-analysis. Oral oncology.

[70] Isles MG, McConkey C, Mehanna HM (2008). A systematic review and meta-analysis of the role of positron emission tomography in the follow up of head and neck squamous cell carcinoma following radiotherapy or chemoradiotherapy. Clin Otolaryngol.

[71] Sheikhbahaei S, Ahn SJ, Moriarty E, Kang H, Fakhry C, Subramaniam RM (2015). Intratherapy or Posttherapy FDG PET or FDG PET/CT for Patients With Head and Neck Cancer: A Systematic Review and Meta-analysis of Prognostic Studies. AJR Am J Roentgenol.

[72] Pak K, Cheon GJ, Nam HY, Kim SJ, Kang KW, Chung JK, Kim EE, Lee DS (2014). Prognostic value of metabolic tumor volume and total lesion glycolysis in head and neck cancer: a systematic review and meta-analysis. J Nucl Med.

[73] Xie P, Li M, Zhao H, Sun X, Fu Z, Yu J (2011). 18F-FDG PET or PET-CT to evaluate prognosis for head and neck cancer: a meta-analysis. J Cancer Res Clin Oncol.

[74] Graham MM, Wahl RL, Hoffman JM, Yap JT, Sunderland JJ, Boellaard R, Perlman ES, Kinahan PE, Christian PE, Hoekstra OS, Dorfman GS (2015). Summary of the UPICT Protocol for 18F-FDG PET/CT Imaging in Oncology Clinical Trials. J Nucl Med.

[75] Boellaard R, Delgado-Bolton R, Oyen WJ, Giammarile F, Tatsch K, Eschner W, Verzijlbergen FJ, Barrington SF, Pike LC, Weber WA, Stroobants S, Delbeke D, Donohoe KJ (2015). FDG PET/CT: EANM procedure guidelines for tumour imaging: version 2.0. Eur J Nucl Med Mol Imaging.

[76] Fukukita H, Suzuki K, Matsumoto K, Terauchi T, Daisaki H, Ikari Y, Shimada N, Senda M (2014). Japanese guideline for the oncology FDG-PET/CT data acquisition protocol: synopsis of Version 2.0. Ann Nucl Med.

[77] de Jong WK, van der Heijden HF, Pruim J, Dalesio O, Oyen WJ, Groen HJ (2007). Prognostic value of different metabolic measurements with fluorine-18 fluorodeoxyglucose positron emission tomography in resectable non-small cell lung cancer: a two-center study. J Thorac Oncol.

[78] Detterbeck FC, Vansteenkiste JF, Morris DE, Dooms CA, Khandani AH, Socinski MA (2004). Seeking a home for a PET, part 3: Emerging applications of positron emission tomography imaging in the management of patients with lung cancer. Chest.

[79] Li YJ, Dai YL, Cheng YS, Zhang WB, Tu CQ (2016). Positron emission tomography (18)F-fluorodeoxyglucose uptake and prognosis in patients with bone and soft tissue sarcoma: A meta-analysis. Eur J Surg Oncol.

[80] Xia Q, Liu J, Wu C, Song S, Tong L, Huang G, Feng Y, Jiang Y, Liu Y, Yin T, Ni Y (2015). Prognostic significance of (18)FDG PET/CT in colorectal cancer patients with liver metastases: a meta-analysis. Cancer Imaging.

[81] Parmar MK, Torri V, Stewart L (1998). Extracting summary statistics to perform meta-analyses of the published literature for survival endpoints. Stat Med.

[82] Tierney JF, Stewart LA, Ghersi D, Burdett S, Sydes MR (2007). Practical methods for incorporating summary time-to-event data into meta-analysis. Trials.

