# Supplementary material for: Prognostic value of 18F-FDG-PET/CT in patients with nasopharyngeal carcinoma: a systematic review and meta-analysis
Source: Oncotarget. 2016 Dec 14;8(20):33884–96. doi: 10.18632/oncotarget.13934 (PMC5464920; doi:10.18632/oncotarget.13934)
Supplement: Supplementary file 1 [file oncotarget-08-33884-s001.pdf]

Prognostic value of ^{18}F-FDG-PET/CT in patients with nasopharyngeal carcinoma: a systematic review and meta-analysis

Supplementary Material

Supplement Table 1. Methodological quality for potentially included studies according to Newcastle–Ottawa scale in this meta-analysis.

1st author [Ref].	Selection (score)	Comparability (score)	Outcome/exposure score	Total (score)
Chan, S. C.[30]	3	1	2	6
Chan, W. K. S.[31]	3	1	2	6
Hsieh, T. C.[32]	3	2	3	8
Hung, T. M.[33]	3	2	3	8
Lee, S. W.[34]	3	1	2	6
Liu, W. S. [35]	3	1	3	7
Moon, S. H.[19]	3	1	3	7
Shen, T.[36]	4	2	3	9
Xiao, W. [17]	3	2	3	8
Xie, P. [37]	3	1	2	6
Yang, Z. [38]	3	1	3	7
Yong, H. I. [39]	3	2	3	8
Yoon, Y. H. [40]	4	2	3	9
Zaghloul, H. A. [41]	3	1	3	7
Zhang, Y. [42]	3	1	2	6

Supplement Table 2. Previous meta-analyses of ^{18}F-FDG PET/CT in patients with head and neck cancer.

Study	Year	Country	No. of studies	No. of patients	Classification	Effect size	Performance measure
Xiao, Y.[57]	2015	China	10	421	Diagnosis	Diagnosis of head and neck carcinoma lesions	Sensitivity/specificity
Rohde, M.[58]	2014	Denmark	9	987	Diagnosis	Diagnosis of head and neck carcinoma lesions	Sensitivity/specificity
Pasamontes Pingarron, J. A.[59]	2008	Espana	19	666	Diagnosis	Diagnosis of head and neck carcinoma lesions	Sensitivity/specificity/likelihood ratios/odds ratios
Cheung, P. K.[68]	2016	Australia	27	1195	Staging	Detecting residual and recurrent disease	Sensitivity/specificity
Sun, R.[60]	2015	China	24	1270	Staging	Detection of regional nodal metastasis	Sensitivity/specificity
Gao, S.[69]	2014	China	10	756	Staging	Detecting residual or recurrent lesion	Sensitivities/specificities/likelihood ratios
Yi, X.[62]	2013	China	17	2912	Staging	Detection of bone metastases	Sensitivities/specificities/likelihood ratios
Yongkui, L.[61]	2013	China	14	742	Staging	Detection of regional nodal metastasis	Sensitivity/specificity
Xu, G[63]	2012	China	8	1147	Staging	Detecting distant malignant metastases	Sensitivity/specificity
Xu, G[64]	2011	China	15	1445	Staging	Diagnosis of distant lesions	Sensitivity/specificity
Xu, G[65]	2011	China	12	1276	Staging	Detecting distant metastases and second primary cancers	Sensitivity/specificity
Gupta, T.[66]	2011	India	51	2335	Staging	Detecting residual and recurrent disease or metastasis	Sensitivity/specificity
Kyzas, P. A.[67]	2008	Greece	32	1236	Staging	Detecting cervical lymph node metastasis	Sensitivities/specificities/likelihood ratios
Isles, M. G.[70]	2008	United Kindom	27	917	Staging	Detecting residual or recurrent lesions after chemoradiotherapy	Sensitivity/specificity
Shelkhbahael, S.[71]	2015	America	26	1708	Prognosis	Prognosis, EFS/OS	Hazard ratios and Mantel-Haenszel risk ratios
Pak, K.[47]	2014	Korea	13	1180	Prognosis	Prognosis, EFS/OS	Hazard ratios
Xie, P.[72]	2011	China	26	1415	Prognosis	Prognosis, DFS/OS/LC	Odds ratios
Zhang, B.[73]	2010	China	8	495	Prognosis	Prognosis, DFS/OS	Risk radios

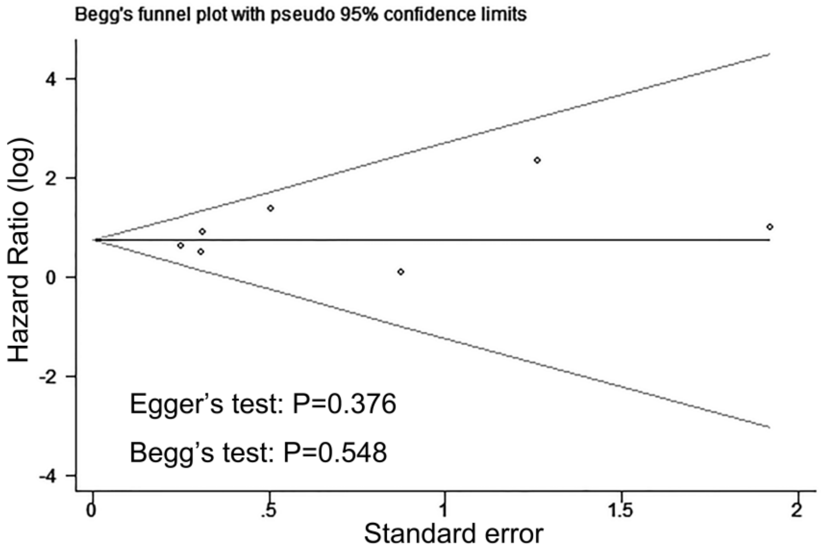

Supplement Figure 1 Publication bias test for the correlations of SUVmax with overall survival. No significant publication bias was detected by Begg's funnel plots (no apparent asymmetry was found) and estimation of P values.

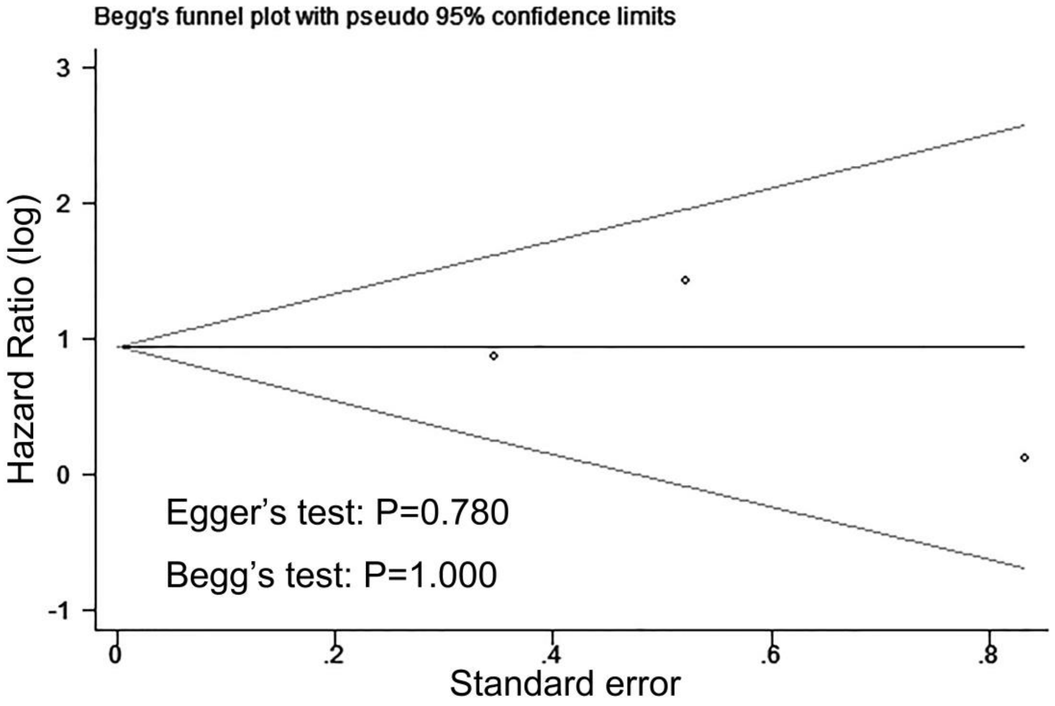

Supplement Figure 2 Publication bias test for the correlations of MTV with event-free survival. No significant publication bias was detected by Begg's funnel plots (no apparent asymmetry was found) and estimation of P values.

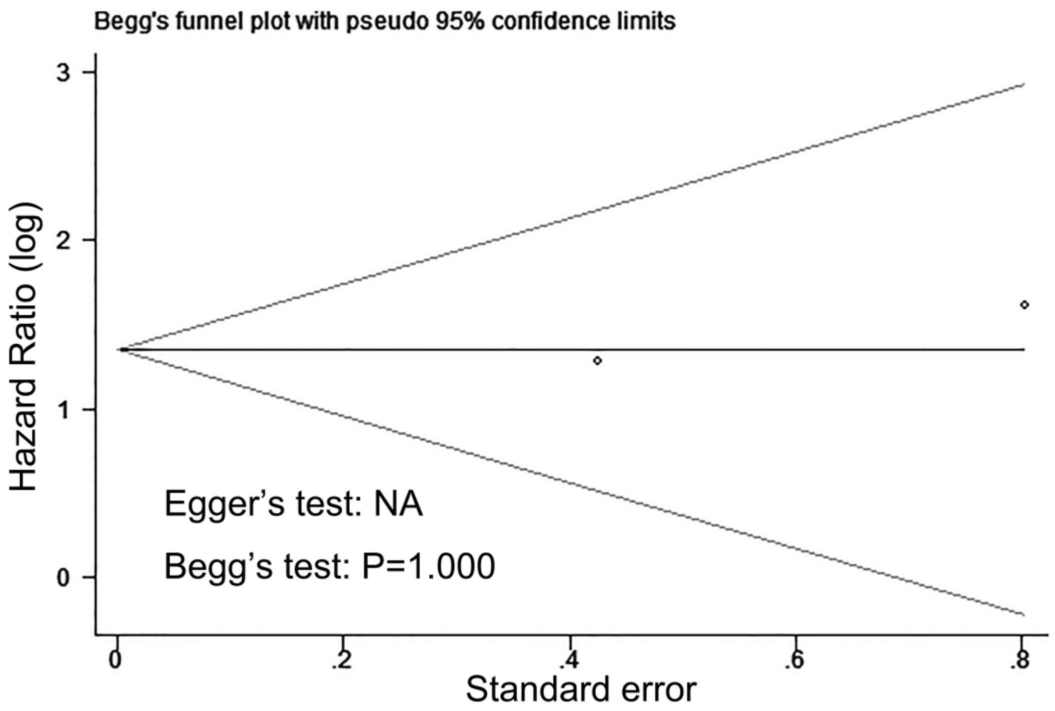

Supplement Figure 3 Publication bias test for the correlations of MTV with overall survival. No significant publication bias was detected by Begg's funnel plots (no apparent asymmetry was found) and estimation of P values.

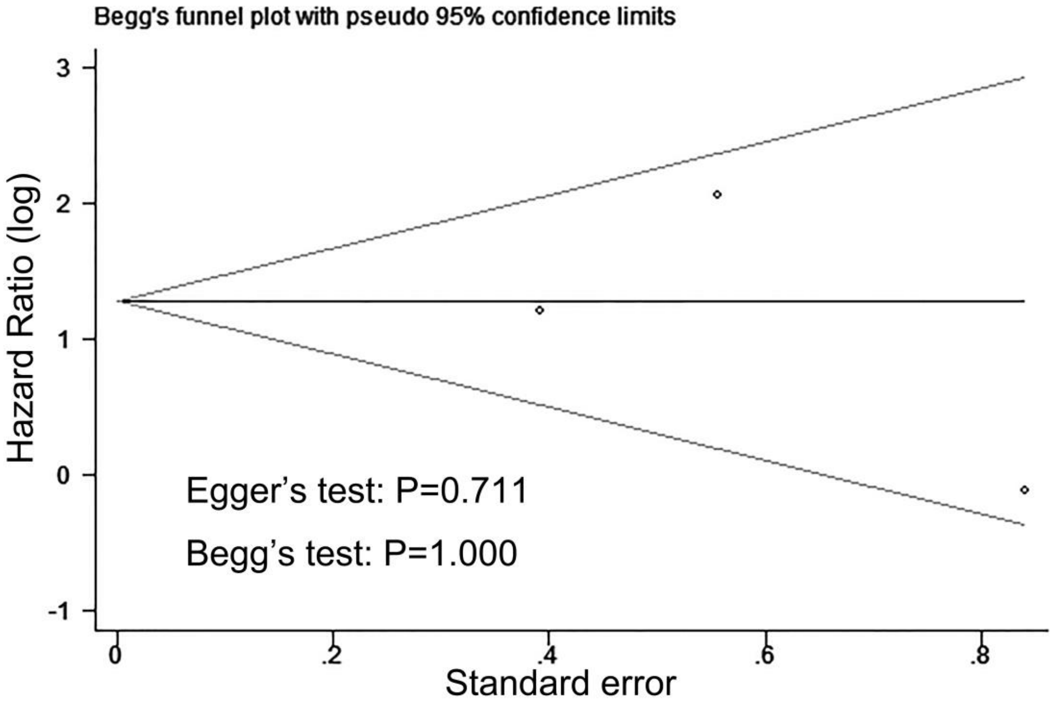

Supplement Figure 4 Publication bias test for the correlations of TLG with event-free survival. No significant publication bias was detected by Begg’s funnel plots (no apparent asymmetry was found) and estimation of P values.

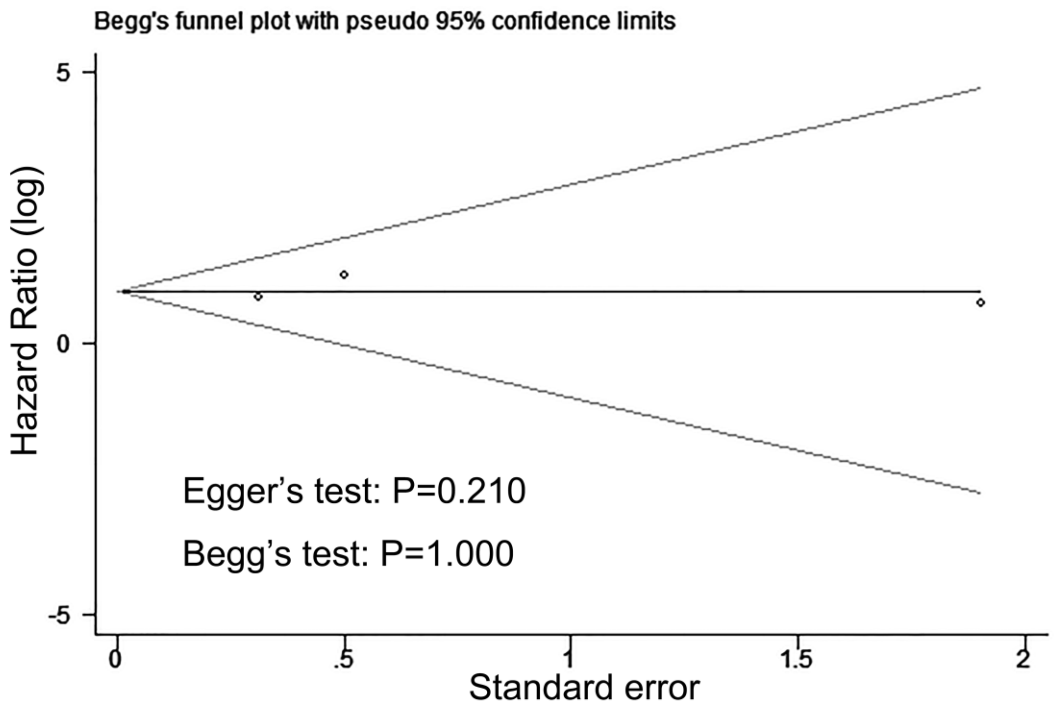

Supplement Figure 5 Publication bias test for the correlations of TLG with overall survival. No significant publication bias was detected by Begg's funnel plots (no apparent asymmetry was found) and estimation of P values.
